# Ambiguities in Powder Indexing: Conjunction of a Ternary and Binary Lattice Metric Singularity in the Cubic System

**DOI:** 10.6028/jres.109.043

**Published:** 2004-12-01

**Authors:** Alan D. Mighell

**Affiliations:** National Institute of Standards and Technology, Gaithersburg, MD 20899-8520

**Keywords:** ambiguities in powder indexing, derivative lattices, *d*-spacings, figure of merit, indexing programs, lattice metric singularity, powder indexing, specialized lattices

## Abstract

A lattice metric singularity occurs when unit cells defining two (or more) lattices yield the identical set of unique calculated *d*-spacings. The existence of such singularities, therefore, has a practical and theoretical impact on the indexing of powder patterns. For example, in experimental practice an indexing program may find only the lower symmetry member of a singularity. Obviously, it is important to recognize such cases and know how to proceed. Recently, we described:

1. a binary singularity involving a monoclinic and a rhombohedral lattice in a subcell-supercell relationship and
2. a second type of singularity—a ternary singularity–in which two of the three lattices are in a derivative composite relationship. In this work, we describe a ternary lattice metric singularity involving a cubic *P*, a tetragonal *P*, and an orthorhombic *C* lattice. Furthermore, there is a binary singularity, involving a hexagonal *P* and orthorhombic *P* lattice, which is characterized by a set of unique *d*-spacings very close to that of the ternary singularity. The existence of such singularities is more common than once thought and requires a paradigm shift in experimental practice. In addition singularities provide opportunities in material design as they point to highly specialized lattices that may be associated with unusual physical properties.

## 1. Introduction

Increasingly, crystal structures are being solved using powder diffraction data. This is due to the evolving power of the *ab initio* structure solving techniques using powder diffraction data and to the fact that in many cases it is impossible to obtain crystals of sufficient size to carry out a single crystal structure analysis. A critical step in the solution process is the determination of a unit cell that defines the lattice. This is commonly done with an indexing program such as DICVOL9l[1] or TREOR[2].

A correct indexing solution is signaled by a high value of the resulting figure of merit (de Wolff[3]; Smith and Snyder[4]). However, a high figure of merit does not guarantee a correct solution. It is a necessary but not sufficient condition for correctness. For example in certain cases, a unique indexing solution does not exist. When a lattice metric singularity occurs [5], there are two or more cells that will account for the same set of observed *d*-spacings. This mathematical condition is defined as follows:

A lattice metric singularity (LMS) occurs when unit cells defining two (or more) lattices yield identical sets of unique calculated *d*-spacings.

Herein singularities in the cubic system will be discussed. In particular special emphasis will be on the conjunction of a ternary and a binary LMS that occurs in the cubic primitive system. This conjunction means that in this system there will always be multiple indexing solutions that are mathematically correct. Consequently, in experimental practice care must be taken to obtain the correct answer. When using indexing procedures, there is no inherent reason to assume the correct answer is necessarily the lattice of highest symmetry. Clearly, in addition to indexing procedures, other methods—e.g., optical, single crystal, etc.,—should be routinely employed to establish uniquely the lattice and symmetry.

Finally it will be shown that the lower symmetry lattices involved in the singularities discussed herein have unusual metric properties and are characterized by specialized reduced forms. A specialized reduced form can signal that certain derivative lattices can have higher symmetry than the original lattice. Accordingly, it is expected the physical properties of actual crystals with such specialized lattices would be influenced by the singularity condition.

## 2. The Ternary Lattice Metric Singularity

The three lattices involved in the ternary singularity are given in Table 1. The lattices I, II, and III are defined by primitive cells 1 and 2, and *C*-centered cell 3, respectively. Alternatively, lattice III can be defined by the primitive reduced cell denoted as 3′. When one compares the volumes of cells 1,2,3′ (i.e., the 3 primitive cells), one notes that the cell volumes are in a 3:1.5:1 relationship. In fact, cells 2 and 3′ are derivative subcells of cell 1.

The reduced forms for cells 1–3 that define the three lattices are given in Table 2. As the reduced forms are all different, the three cells clearly define different lattices. The reduced forms 3, 11 and 38 are characteristic of a primitive cubic, primitive tetragonal, and a *C*-centered orthorhombic lattice, respectively. Detailed inspection of the second two reduced forms shows that there is more specialization than required for the given reduced form type. For example, in the case of lattice II, the 1:1:2 relationship between the symmetrical dot products—***a·a*:*b·b*:*c·c***—of the reduced form implies a highly specialized lattice.

In Table 3, we present the unique *d*-spacings for the three lattices. The sets of unique interplanar spacings are identical. However, the columns labeled *M* for lattices I–III show that the number of *d*-spacings with a given calculated *d*-value can be different. Consider a calculated *d*-value equal to 1.2910 Å (Note: 1 Å [= 0.1 nm] is the common unit in crystallography). For this value the table shows that the numbers calculated for lattices I, II, and III are 1, 2 and 7, respectively. When the program NBS*AIDS83[8] calculates more than one (not symmetrically related) *d*-spacing with the same value, the *hkl* indices for only the first of the group are given in Table 3.

For the nonspecialized lattice of tetragonal or orthorhombic symmetry, the program would calculate *M* discrete *d*-spacings for those cases in which *M* > 1 in Table 3. Inspection of *d*-spacing data for lattices II and III reveals that these two lattices are highly specialized in the sense that the value of *M* is often greater than 1. Thus the patterns have far fewer discrete lines than normally possible for the given symmetry. This is shown in Table 4. In column four of this table, the compression ratio is given which is the ratio of the unique *d*-spacings to the total calculated *d*-spacings. For the tetragonal lattice, the compression ratio is 0.422 when the *d*-spacings are calculated out to a 2*θ* of 55° with *λ* = 0.7093 Å.

## 3. The Binary Lattice Metric Singularity

The two lattices involved in the binary singularity are given in Table 5. These lattices are defined by primitive cells 4 and 5. Cell 4 defines an orthorhombic lattice whereas cell 5 of twice the volume defines a hexagonal lattice. The nature of the lattice relationship can be deduced from the transformation matrix relating the two cells. Thus lattice V is a superlattice of lattice IV.

The reduced forms for cells 1 and 2 defining the two lattices are given in Table 6. As the reduced forms are different, the two cells clearly define different lattices. The reduced form type 32 is characteristic for a primitive orthorhombic lattice and reduced form 22 for a hexagonal lattice. Detailed inspection of each reduced form (or normalized reduced form) shows that there is more specialization than required for the given reduced form type. For example, in the case of lattice IV, the 1:1.5:3 relationship between the symmetrical dot products—***a·a*:*b·b*:*c·c***—of the reduced form implies a highly specialized lattice.

In Table 7, we present the unique *d*-spacings for the two lattices. The sets of unique interplanar spacings are identical. However, the columns labeled *M* for lattices IV and V show that the number of *d*-spacings with a given calculated *d*-value can be different. Consider a calculated *d*-value equal to 1.3056. For this value, the table shows that the numbers calculated for lattices IV and V are 4 and 2, respectively. When the program NBS*AIDS83[8] calculates more than one (not symmetrically related) *d*-spacing with the same value, the *hkl* indices for only the first of the group are given in the table.

For a nonspecialized lattice of orthorhombic or hexagonal symmetry, the program would calculate *M* discrete *d*-spacings for those cases in which *M* > 1 in Table 7. Inspection of the two patterns reveals that these two lattices are highly specialized in the sense that the value of *M* is often greater than 1. Thus the patterns have far fewer discrete lines than normally possible for the given symmetry. This is shown in Table 8. In column 4 of this table, the compression ratio is given which is the ratio of the unique *d*-spacings to the total calculated *d*-spacings. For the orthorhombic lattice, the compression ratio is 0.29 when the *d*-spacings are calculated out to a 2*θ* of 55° with *λ* = 0.7093 Å.

## 4. Conjunction of Lattice Metric Singularities in the Cubic *P* System

The singularities discussed above can present difficulties in indexing powder patterns. As an example, let us assume that an indexing program is presented with a set of observed *d*-spacings obtained from a crystal whose lattice is correctly defined by a cubic primitive unit cell. What should the indexing program reveal? Mathematically, a unique indexing solution does not exist. As the data in Table 3 illustrate, three distinct lattices—defined by a cubic *P*, a tetragonal *P*, and an orthorhombic *C* unit cell—are characterized by the same set of unique *d*-spacings. Further complicating this situation is the fact that this ternary LMS is in conjunction with a binary LMS, i.e., the two lattices in the binary LMS (Table 7) are characterized by a set of *d*-spacings that is almost the same as the one in the ternary LMS (Table 3).

This conjunction is demonstrated in Table 9 which shows how closely the sets of *d*-spacings in the two singularities are related. Consider a data set comprised of the first 20 possible *d*-spacings for the cubic *P* crystal. In addition assume that merely one *d*-spacings [2.7386] is accidentally absent. In this case there are five possible answers that are mathematically correct! In fact, using this data set as input to the Boultif and Louër indexing program [1], it was possible to obtain all five answers.

## 5. Discussion

The results above are described in terms of a lattice defined by a cubic primitive cell (*a* = 8.6603 Å). However, by the principle of similarity, the same ambiguities in indexing exist with respect to experimental data determined from any crystal characterized by a cubic primitive cell. Furthermore, as the cubic primitive lattice is common for inorganic materials, the above type of ambiguity can often present difficulties in common practice. This is especially true today as more and more structures are solved from powder data only.

As demonstrated above, there is more than one mathematically correct answer when indexing “ideal” data from a cubic primitive crystal. In experimental practice, however, the situation is more complex as the quality of the observed data is influenced by such factors as experimental errors, accidental absences, and impurities. Consequently the indexing program may not yield all the potential answers and it may not yield the correct answer! This happened to us in the course of our experimental work. In our case, we obtained only the orthorhombic solution in the binary singularity when, in fact, the crystal was later shown to be cubic primitive. Other scenarios are equally possible. For example, if one obtains only the primitive tetragonal or *C*-centered orthorhombic cell of the ternary singularity, then one would have a derivative subcell of the correct lattice. Subsequent structure solving techniques may then yield an incorrect solution using the derivative cell as the basis cell.

How can errors in lattice determination be prevented? One key to prevention is to inspect the reduced form. A warning flag is extra specialization in the reduced form. As Table 2 and 6 show, the reduced forms for the lower symmetry indexing answers all have more specialization than required for the given reduced form type [7]. For example, in Table 2 the symmetrical scalars of the reduced form (***a·a b·b* c·*c***) for lattice II are in the ratio 1:1:2 (whereas the requirement is simply 1:1:1+x). Extra specialization of this nature commonly indicates that something is unusual such as an incorrect answer or a highly specialized lattice. Another key to error prevention is to use other methods along with powder indexing. For example, any primitive cell determined via the precession method would distinguish between the potential indexing solutions. Likewise, optical techniques, such as polarization microscopy, would be helpful in determining the correct lattice symmetry. Finally, the crystallographic databases should be routinely searched for the same and related materials and to orient the crystal under study with extant materials.

The above discussion has focused on the cubic crystals characterized by a primitive lattice. However, for cubic crystals, mathematical ambiguities in indexing are not confined to crystals characterized by a cubic primitive lattice. They also occur in the centered cubic lattices. In the cubic *F* system, there is a ternary lattice metric singularity and in the cubic I system there is a quaternary lattice metric singularity [9]. Table 10 presents the four lattices in the quaternary LMS. Inspection of Table 10 reveals that the Lattices II–IV are derivative sublattices of lattice I. The volume volume ratios for the four reduced cells for lattices I–IV are 1:1/2:1/3:1/4. As in the ternary LMS in the cubic *P* system, the reduced forms for lattices I–IV have more specialization than required for the given reduced form type.

## 6. Conclusion

The above analysis shows that lattice metric singularities are inherent in the cubic system. In the Cubic *P*, *I* and *F* systems, we encounter a ternary (in conjunction with a binary), a quaternary, and a ternary lattice metric singularity, respectively. Singularities are a mathematical property of lattices which cannot be ignored. Consequently, one cannot prove that a crystal is cubic by indexing procedures alone.

Obviously, it is important to be aware of all members of a singularity. Due to a variety of factors (i.e., quality of data, accidental absences, quality of crystal, etc.), indexing programs may miss some of the members of a given singularity. In the above discussion, it was noted that in experimental practice, it is possible to miss the highest symmetry member of a singularity.

When a singularity occurs, which member represents the correct solution? Usually one would expect that the indexing solution with the highest symmetry is the correct solution. But is this a valid assumption? Have certain crystals inadvertently and unknowingly been assigned an erroneous higher crystal symmetry? Alternatively, if a crystal is indexed on the basis of a lower symmetry lattice, is this a valid assumption? The following example illustrates an actual case.

### 6.1 Singularities in Experimental Practice—OsO_2_

Knowledge of singularities is important in experimental practice. Thus one should be aware when one is dealing with a member of a singularity. For example, a case of especial interest is OsO_2_ which has been reported [10–13] to be a rutile type structure crystallizing in the tetragonal system. An analysis of the reported cell dimensions shows that this lattice is involved in the type of quaternary singularity shown above in Table 10. In fact, the data reported in the Powder Diffraction File [12] (PDF no 43-1044) can be refined to yield a cubic *I*-centered cell [*a* = 6.36553(2) Å] with an excellent figure of merit [M(20) = 178, F(20) = 77)]. Likewise the other two members of this quaternary singularity can also be refined to yield a high figure of merit.

In understanding the physical properties of this material, the fact that OsO_2_ is reported as the tetragonal member of a quaternary singularity has practical and theoretical consequences. First, was an error made in symmetry determination? Was it incorrect to assume that this compound is tetragonal like all the other rutile-related structures (see Table II of Rogers, et al. [11]? Most likely an error was not made as Boman [13] has carried out a “precision determination” of the tetragonal crystal structure based on single crystal techniques. Second, if the tetragonal cell and space group are indeed correct, there are interesting implications. This means that the lattice of this compound, in contrast to all the other rutile type structures, is special as it is a rutile structure involved in a singularity. The OsO_2_ crystal would have a superlattice of higher symmetry and be characterized by a powder pattern with far fewer unique *d*-spacings than related rutile-type materials.

### 6.2 Singularities Offer Opportunities

Lattice metric singularities offer novel opportunities. They provide a mechanism to evaluate powder indexing programs. If working properly, an indexing program should obtain all members of a singularity. From the database building perspective, they provide a mathematical mechanism to evaluate certain types of lattices which are defined by specialized reduced cells. And from the synthetic perspective, their existence makes it possible to prepare compounds with potentially unusual physical properties.

## Figures and Tables

**Table 1:** The three lattices involved in a ternary lattice metric singularity^a^. The unique sets of calculated *d*-spacings for the three lattices are identical

	Lattice I: Cubic *P*	Lattice II: Tetragonal *P*	Lattice III: Orthorhombic *C*	Lattice III: Reduced *P*
	Cell 1	Cell 2	Cell 3	Cell 3′
*a* (Å)	8.660254	6.123724	4.082483	4.082483
*b* (Å)	8.660254 6.123724		12.247449	6.454972
*c* (Å)	8.660254	8.660254	8.660254	8.660254
*a* (°)	90.0	90.0	90.0	90.0
*β* (°)	90.0	90.0	90.0	90.0
*γ* (°)	90.0	90.0	90.0	108.435
*V*(Å3)	649.52 324.76		433.01	216.51
*c/a*		1.4142	2.1213	2.1213
*c/b*			0.7071	1.3416

a Lattice relationships:
$$\begin{array}{ll} \text{Cell}\;1\to \text{Cell}\;2 & T=[\begin{array}{lllllllllll} \;0 & -1/2 & \;1/2 & / & 0 & 1/2 & 1/2 & / & -1 & \;0 & \;0\; \end{array}]\\ \text{Cell}\;1\to \text{Cell}\;3 & T=[\begin{array}{lllllllllll} \;0 & -1/3 & 1/3 & / & \;0 & \;1 & \;1 & \;/ & \;-1 & \;0 & \;0\; \end{array}]\\ \text{Cell}\;2\to \text{Cell}\;3 & T=[\begin{array}{lllllllllll} \;0 & -2/3 & \;0 & \;/ & -2 & \;0 & \;0 & \;/ & \;0 & \;0 & -1\; \end{array}] \end{array}$$
$$\begin{array}{ll} \text{Cell}\;3\prime \to \text{Cell}\;1 & T=[\begin{array}{lllllllllll} \;2 & 1 & 0 & / & -1 & 1 & 0 & / & 0 & 0 & 1\; \end{array}]\\ \text{Cell}\;2\to \text{Cell}\;1 & T=[\begin{array}{lllllllllll} -1 & 1 & 0 & / & \;0 & 0 & 1 & / & 1 & 1 & 0\; \end{array}] \end{array}$$ NIST*LATTICE [6] was used to determine the above and other lattice relationships cited herein.

**Table 2:** Reduced form data for cells 1–3^a^ defining Lattices I–III. The reduced forms for the tetragonal *P* and the orthorhombic *C* lattices have extra specialization

	Lattice I: Cubic *P*			Lattice II: Tetragonal *P*			Lattice III: Orthorhombic *C*		
Reduced form number		3			11			38	
Reduced form definition^b^	***a·a***	***a·a***	***a·a***	***a·a***	***a·a***	***c·c***	***a·a***	***b·b***	***c·c***
	0	0	0	0	0	0	0	0	***−a·a***/2
Reduced form (Å^2^)	75.0	75.0	75.0	37.5	37.5	75.0	16.667	41.667	75.0
	0	0	0	0	0	0	0	0	−8.331
Reduced form normalized	1	1	1	1	1	2	1	2.5	4.5
	0	0	0	0	0	0	0	0	−0.5

a Cell dimensions for cells 1–3 are given in Table 1.

b See metric classification of the 44 reduced forms given in Table 2 of Ref.[7].

**Table 3:** Ternary lattice metric singularity. The values of the calculated *d*-spacings (Å) for the three lattices are identical

No.	Lattice I: Cubic *P*^a^					Lattice II: Tetragonal *P*^b^					Lattice III: Orthorhombic *C*^c^				
	*h k l*			*d*-calc	*M* d	*h k l*			*d*-calc	*M*	*h k l*			*d*-calc	*M*
1	1	0	0	8.6603	1	0	0	1	8.6603	1	0	0	1	8.6603	1
2	1	1	0	6.1237	1	1	0	0	6.1237	1	0	2	0	6.1237	1
3	1	1	1	5.0000	1	1	0	1	5.0000	1	0	2	1	5.0000	1
4	2	0	0	4.3301	1	1	1	0	4.3301	2	0	0	2	4.3301	1
5	2	1	0	3.8730	1	1	1	1	3.8730	1	1	1	0	3.8730	1
6	2	1	1	3.5355	1	1	0	2	3.5355	1	1	1	1	3.5355	2
7	2	2	0	3.0619	1	2	0	0	3.0619	2	0	4	0	3.0619	1
8	3	0	0	2.8868	1	0	0	3	2.8868	2	1	3	0	2.8868	4
9	3	1	0	2.7386	1	2	1	0	2.7386	1	1	3	1	2.7386	1
10	3	1	1	2.6112	1	2	1	1	2.6112	2	0	2	3	2.6112	1
11	2	2	2	2.5000	1	2	0	2	2.5000	1	0	4	2	2.5000	1
12	3	2	0	2.4019	1	1	1	3	2.4019	1	1	3	2	2.4019	1
13	3	2	1	2.3146	1	2	1	2	2.3146	1	1	1	3	2.3146	1
14	4	0	0	2.1651	1	2	2	0	2.1651	2	0	0	4	2.1651	1
15	4	1	0	2.1004	1	2	2	1	2.1004	2	1	5	0	2.1004	2
16	3	3	0	2.0412	1	3	0	0	2.0412	2	0	6	0	2.0412	5
17	3	3	1	1.9868	1	3	0	1	1.9868	2	0	6	1	1.9868	2
18	4	2	0	1.9365	1	3	1	0	1.9365	3	2	2	0	1.9365	1
19	4	2	1	1.8898	1	3	1	1	1.8898	1	2	2	1	1.8898	3
20	3	3	2	1.8464	1	3	0	2	1.8464	1	0	6	2	1.8464	2
21	4	2	2	1.7678	1	3	1	2	1.7678	2	2	2	2	1.7678	2
22	5	0	0	1.7321	1	0	0	5	1.7321	2	0	0	5	1.7321	2
23	5	1	0	1.6984	1	3	2	0	1.6984	2	2	4	0	1.6984	2
24	5	1	1	1.6667	1	3	2	1	1.6667	3	2	4	1	1.6667	4
25	5	2	0	1.6082	1	1	1	5	1.6082	2	1	7	0	1.6082	2
26	5	2	1	1.5811	1	3	2	2	1.5811	1	1	7	1	1.5811	3
27	4	4	0	1.5309	1	4	0	0	1.5309	2	0	8	0	1.5309	1
28	5	2	2	1.5076	1	2	0	5	1.5076	2	0	8	1	1.5076	4
29	5	3	0	1.4852	1	4	1	0	1.4852	2	0	6	4	1.4852	3
30	5	3	1	1.4639	1	4	1	1	1.4639	3	2	4	3	1.4639	1
31	6	0	0	1.4434	1	3	3	0	1.4434	4	2	6	0	1.4434	4
32	6	1	0	1.4237	1	3	3	1	1.4237	1	2	6	1	1.4237	1
33	6	1	1	1.4049	1	4	1	2	1.4049	2	1	7	3	1.4049	2
34	6	2	0	1.3693	1	4	2	0	1.3693	3	2	6	2	1.3693	1
35	6	2	1	1.3525	1	4	2	1	1.3525	3	0	8	3	1.3525	3
36	5	4	1	1.3363	1	3	2	4	1.3363	1	3	1	1	1.3363	3
37	5	3	3	1.3207	1	4	1	3	1.3207	2	0	6	5	1.3207	2
38	6	2	2	1.3056	1	4	2	2	1.3056	2	0	4	6	1.3056	1
39	6	3	0	1.2910	1	3	3	3	1.2910	2	1	9	0	1.2910	7
40	6	3	1	1.2769	1	2	1	6	1.2769	1	1	9	1	1.2769	2

a Cell 1 (Cubic *P*):    *a* = 8.660254 Å,  *V* = 649.52 Å^3^.

b Cell 2 (Tetragonal *P*):  *a* = 6.123724 Å,  *c* = 8.660254 Å,  *V* = 324.76 Å^3^.

c Cell 3 (Orthorhombic *C*): *a* = 4.082483 Å,  *b* = 12.247449Å  *c* = 8.660254 Å,  *V* = 433.01 Å^3^.

d Number of lines calculated (NBS*AIDS83[8]) with the specified *d*-spacing value.

**Table 4:** Ternary lattice metric singularity. The *d*-spacings for each lattice were calculated^a^ using the specified 2θ maximum values and *λ* = 0.7093 Å. The number of unique *d*-spacings for the three lattices is identical. The low values for the compression ratios for lattices II and III show that they are specialized (i.e., many *d*-spacings have the same value)

	2θ Maximum	Unique *d*-spacings	Total *d*-spacings	Compression Ratio^b^
	40	59	59	1
Cell 1^c^	45	74	74	1
Lattice I	50	90	90	1
	55	106	106	1
	40	59	117	0.504
Cell 2^d^	45	74	157	0.471
Lattice II	50	90	202	0.446
	55	106	251	0.422
	40	59	140	0.421
Cell 3^e^	45	74	189	0.392
Lattice III	50	90	251	0.359
	55	106	322	0.329

a NBS*AIDS83[8].

b Compression ratio = “unique *d*-spacings / possible *d*-spacings” for a given symmetry.

c Cell 1 (Cubic *P*):    *a* = 8.660254 Å,  *V* = 649.52 Å^3^.

d Cell 2 (Tetragonal *P*):  *a* = 6.123724 Å,  *c* = 8.660254 Å,  V = 324.76 Å^3^.

e Cell 3 (Orthorhombic *C*): *a* = 4.082483 Å,  *b* = 12.247449 Å,  c = 8.660254 Å,  *V* = 433.01 Å^3^.

**Table 5:** The two lattices involved in a binary lattice metric singu-larity^a^. The unique sets of calculated *d*-spacings for the two lattices are identical

	Lattice IV: Orthorhombic *P*	Lattice V: Hexagonal *P*
	Cell 4	Cell 5
*a*(Å)	5.0	10.0
*b*(Å)	6.123724	10.0
*c*(Å)	8.660254	6.123724
*α* (°)	90.0	90.0
*β* (°)	90.0	90.0
*γ* (°)	90.0	120.0
*V*(Å^3^)	265.17	530.33
*c/a*	1.7320	0.6124
*c/b*	1.4142	

a Lattice relationships:
$$\begin{array}{ll} \text{Cell}\;4\to \text{Cell}\;5 & T= \end{array}[\begin{array}{lllllllllll} \;2 & 0 & 0 & / & -1 & 0 & -1 & / & 0 & 1 & 0\; \end{array}]$$ NIST*LATTICE[6] was used to determine these and other lattice relationships cited herein.

**Table 6:** Reduced form data for cells 4–5^a^ defining Lattices IV–V. Both reduced forms have extra specialization

	Lattice IV: Orthorhombic *P*			Lattice V: Hexagonal *P*		
Reduced form number		32			22	
Reduced form definition^b^	***a·a***	***b·b***	***c·c***	***a·a***	***b·b***	***b·b***
	0	0	0	−***b·b/*** 2	0	0
Reduced form (Å^2^)	25.0	37.5	75.0	37.5	100	100
	0	0	0	−50	0	0
Reduced form normalized	1	1.5	3	1	2.67	2.67
	0	0	0	−1.33	0	0

a Cell dimensions for cells 4–5 are given in Table 5.

b See metric classification of the 44 reduced forms given in Table 2 of Ref.[7].

**Table 7:** Binary lattice metric singularity. The values of the calculated *d*-spacings for the two lattices are identical

No.	Lattice I: Orthorhombic^a^					Lattice II: Hexagonal b				
	*h k l*			*d*-calc	*M* c	*h k l*			*d*-calc	*M*
1	0	0	1	8.6603	1	1	0	0	8.6603	1
2	0	1	0	6.1237	1	0	0	1	6.1237	1
3	1	0	0	5.0000	2	1	0	1	5.0000	2
4	1	0	1	4.3301	2	2	0	0	4.3301	1
5	1	1	0	3.8730	1	1	1	1	3.8730	1
6	1	1	1	3.5355	2	2	0	1	3.5355	1
7	1	0	2	3.2733	1	2	1	0	3.2733	1
8	0	2	0	3.0619	1	0	0	2	3.0619	1
9	0	0	3	2.8868	3	3	0	0	2.8868	3
10	1	2	0	2.6112	2	3	0	1	2.6112	2
11	2	0	0	2.5000	4	2	0	2	2.5000	2
12	2	0	1	2.4019	1	3	1	0	2.4019	1
13	2	1	0	2.3146	2	2	2	1	2.3145	1
14	2	1	1	2.2361	2	3	1	1	2.2361	2
15	2	0	2	2.1651	2	4	0	0	2.1651	1
16	0	2	3	2.1004	1	3	0	2	2.1004	1
17	0	3	0	2.0412	3	4	0	1	2.0412	2
18	0	3	1	1.9868	2	3	2	0	1.9868	2
19	2	2	0	1.9365	2	2	2	2	1.9365	1
20	1	3	0	1.8898	4	1	1	3	1.8898	4
21	0	3	2	1.8464	2	2	0	3	1.8464	1
22	2	1	3	1.8058	1	4	1	1	1.8058	1
23	2	2	2	1.7678	2	4	0	2	1.7678	1
24	0	0	5	1.7321	2	5	0	0	1.7321	2
25	3	0	0	1.6667	4	5	0	1	1.6667	4
26	1	0	5	1.6366	3	4	2	0	1.6366	1
27	3	1	0	1.6082	2	3	3	1	1.6082	2
28	2	3	0	1.5811	5	4	2	1	1.5811	2
29	2	3	1	1.5554	2	5	1	0	1.5554	2
30	0	4	0	1.5309	1	0	0	4	1.5309	1
31	3	1	2	1.5076	3	5	1	1	1.5076	3
32	2	3	2	1.4852	2	4	0	3	1.4852	1
33	1	4	0	1.4639	2	1	1	4	1.4639	2
34	3	0	3	1.4434	7	6	0	0	1.4434	3
35	1	3	4	1.4237	2	4	3	0	1.4237	2
36	3	1	3	1.4049	2	6	0	1	1.4049	1
37	3	2	2	1.3868	5	5	2	0	1.3868	5
38	1	1	6	1.3525	2	3	0	4	1.3525	2
39	3	0	4	1.3207	2	6	1	0	1.3207	2
40	2	4	0	1.3056	4	6	0	2	1.3056	2

a Cell 4 (Orthorhombic *P*): *a* = 5.0 Å,   *b* = 6.123724 Å,  *c* = 8.660254 Å,  *V* = 265.17 Å^3^.

b Cell 5 (Hexagonal *P*):  *a* = 10.0 Å, *c* = 6.123724 Å,  *V* = 530.33 Å^3^.

c Number of lines calculated (NBS*AIDS83[8]) with the specified *d*-spacing value.

**Table 8:** Binary lattice metric singularity. The *d*-spacings for each lattice were calculated^a^ using the specified 2θ maximum values and *λ* = 0.7093 Å. The number of unique *d*-spacings for the two lattices is identical. The low values for the compression ratios for lattices IV and V show that they are special (i.e., many *d*-spacings have the same value)

	2θ Maximum	Unique *d*-spacings	Total *d*-spacings	Compression Ratio^b^
	40	64	174	0.368
Cell 4^c^	45	81	242	0.335
Lattice IV	50	97	306	0.317
	55	117	400	0.292
	40	64	126	0.508
Cell 5^d^	45	81	171	0.474
Lattice V	50	97	215	0.451
	55	117	275	0.425

a NBS*AIDS83[8].

b Compression ratio = “unique *d*-spacings/possible *d*-spacings” for a given symmetry.

c Cell 4 (Orthorhombic *P*): *a* = 5.0 Å,  *b* = 6.123724 Å,  *c* = 8.660254 Å,  *V* = 265.17 Å^3^.

d Cell 5 (Hexagonal *P*):  *a* = 10.0 Å,  *c* = 6.123724 Å,  *V* = 530.33 Å^3^.

**Table 9:** Conjunction of a Ternary (Lattices I, II, III) and a Binary (Lattices IV and V) Lattice Metric Singularity. The sets of calculated *d*-spacings (Å) for the lattices in the ternary (I, II, III) and binary (IV, V) singularities are almost identical

No	Lattice I: Cubic *P*^a^	Lattice II: Tetragonal *P*^b^	Lattice III: Orthorhombic *C*^c^	Lattice IV: Orthorhombic *P*^d^	Lattice V: Hexagonal *P*^e^
	*d*-calc	*d*-calc	*d*-calc	*d*-calc	*d*-calc
1	8.6603	8.6603	8.6603	8.6603	8.6603
2	6.1237	6.1237	6.1237	6.1237	6.1237
3	5.0000	5.0000	5.0000	5.0000	5.0000
4	4.3301	4.3301	4.3301	4.3301	4.3301
5	3.8730	3.8730	3.8730	3.8730	3.8730
6	3.5355	3.5355	3.5355	3.5355	3.5355
7				3.2733	3.2733
8	3.0619	3.0619	3.0619	3.0619	3.0619
9	2.8868	2.8868	2.8868	2.8868	2.8868
10	2.7386	2.7386	2.7386		
11	2.6112	2.6112	2.6112	2.6112	2.6112
12	2.5000	2.5000	2.5000	2.5000	2.5000
13	2.4019	2.4019	2.4019	2.4019	2.4019
14	2.3146	2.3146	2.3146	2.3146	2.3145
15				2.2361	2.2361
16	2.1651	2.1651	2.1651	2.1651	2.1651
17	2.1004	2.1004	2.1004	2.1004	2.1004
18	2.0412	2.0412	2.0412	2.0412	2.0412
19	1.9868	1.9868	1.9868	1.9868	1.9868
20	1.9365	1.9365	1.9365	1.9365	1.9365
21	1.8898	1.8898	1.8898	1.8898	1.8898
22	1.8464	1.8464	1.8464	1.8464	1.8464

a Cell 1 (Cubic *P*):    *a* = 8.660254 Å,  *V* = 649.52 Å3.

b Cell 2 (Tetragonal *P*):  *a* = 6.123724 Å,  *c* = 8.660254 Å,  *V* = 324.76 Å^3^.

c Cell 3 (Orthorhombic *C*): *a* = 4.082483 Å,  *b* = 12.247449 Å, *c* = 8.660254 Å,  *V* = 433.01 Å^3^.

d Cell 4 (Orthorhombic *P*): *a* = 5.0 Å,    *b* = 6.123724 Å,  *c* = 8.660254 Å,  *V* = 265.17 Å^3^.

e Cell 5 (Hexagonal *P*):  *a* = 10.0 Å,   *c* = 6.123724 Å,  *V* = 530.33 Å^3^.

**Table 10:** Quaternary lattice metric singularity. The four lattices yield the same set of unique calculated *d*-spacings. For each lattice the table gives the conventional cell along with the corresponding reduced cell and normalized reduced form

	Lattice I: Cubic *I*	Lattice II: Tetragonal *P*	Lattice III: Orthorhombic *F*	Lattice IV: Orthorhombic *P*

Conventional Cells				
Cell	Cell 1	Cell 2	Cell 3	Cell 4
*a*(Å)	8.6603	6.1237	4.0825	3.0619
b(Å)	8.6603	6.1237	8.6603	4.3301
*c*(Å)	8.6603	4.3301	12.2475	6.1237
*α* (°)	90.0	90.0	90.0	90.0
*β* (°)	90.0	90.0	90.0	90.0
*γ* (°)	90.0	90.0	90.0	90.0
*V*(Å^3^)	649.52	162.38	433.01	81.19
*a/c*	1.0	$\sqrt{2}$	1/3	1/2
*b/c*	1.0	$\sqrt{2}$	12	12

Reduced Cells				
Cell	R1	R2^a^	R3^b^	R4^c^

*a*(Å)	7.5000	4.3301	4.0825	3.0619
*b*(Å)	7.5000	6.1237	4.7871	4.3301
*c*(Å)	7.5000	6.1237	6.4550	6.1237
*α* (°)	109.471	90.0	82.251	90.0
*β* (°)	109.471	90.0	71.565	90.0
*γ* (°)	109.471	90.0	64.761	90.0
*V*(Å^3^)	324.76	162.38	108.25	81.19

Normalized Reduced Forms				
Form	F1	F2	F3	F4

***a·a***	1	1	1	1
***b·b***	1	2	1.375	2
***c·c***	1	2	2.500	4
***b·c***	−1/3	0	1/4	0
***a·c***	−1/3	0	1/2	0
***a·b***	−1/3	0	1/2	0
Form No.	5	21	26	32

Transformations

a R2 → R1 [ 1 −1 0 / −1 0 1 / −1 0 −1 ] ∆ = 2.

b R3 → R1 [ 1 1 0 / −2 1 0 / 0 −1 1 ] ∆ = 3.

c R4 → R1 [ 0 −1 −1 / 2 1 0 / 0 −1 1 ] ∆ = 4.
